# Glistening formation in a new hydrophobic acrylic intraocular lens

**DOI:** 10.1186/s12886-020-01430-z

**Published:** 2020-05-06

**Authors:** Timur M. Yildirim, Hui Fang, Sonja K. Schickhardt, Qiang Wang, Patrick R. Merz, Gerd U. Auffarth

**Affiliations:** 1 The David J. Apple International Laboratory for Ocular Pathology, Department of Ophthalmology, University of Heidelberg, Im Neuenheimer Feld 400, 69120 Heidelberg, Germany; 2 Department of Ophthalmology, The Third Affiliated Hospital of Wenzhou Medical University, 108# Wansong Road, Rui’an, Zhejiang, 325200 China

**Keywords:** Glistenings, Hydrophobic acrylic, IOL material quality, IOL pathology, IOL material change, AcrySof, Eyecryl, IOL aging

## Abstract

**Background:**

The formation of fluid-filled microvacuoles, termed glistenings, is a common complication of intraocular lenses (IOLs) made from hydrophobic acrylate. Using our well-established in-vitro laboratory method, we evaluated a new IOL material’s resistance to glistening formation.

**Methods:**

An in-vitro stress test for glistening induction was performed on 20 samples of hydrophobic acrylic IOLs: ten of the new Eyecryl ASHFY600 (Biotech Vision Care, Ahmedabad, India) compared with ten samples of AcrySof IQ SN60WF (Alcon, Fort Worth, USA). The number of microvacuoles per square millimetre (MV/mm^2^) was evaluated in five sections of each IOL. The results for each model were compared and rated on a modified Miyata Scale for grading glistening severity.

**Results:**

In all cases, glistening number was higher in the central section of the IOL optic than in the periphery. Mean number of MV/mm^2^ was highest in the central part of the AcrySof IQ SN60WF, with 41.84 (±27.67) MVs/mm^2^. The lowest number of glistenings was found in the five sections of the Eyecryl ASHFY600 with 0.52 (±0.24) MVs/mm^2^. Mean value of the Eyecryl ASHFY600 IOL, using the Miyata Scale, was Zero.

**Conclusion:**

In this in-vitro laboratory study, the new hydrophobic acrylic IOL showed a high resistance to microvacuole formation. Results from this in-vitro study suggest that glistening numbers will be low in clinical use in the Eyecryl ASHFY600.

## Background

Hydrophobic acrylic intraocular lenses (IOLs) can develop a whitish, opaque material change under certain environmental conditions or over time [1, 2]. This appearance is caused by fluid-filled microvacuoles, so called glistenings, that were first described in 1984 [3]. Early hydrophobic acrylate IOL materials were composed of copolymers that allowed low equilibrium water content of below 1%. In these materials, water that enters the polymer, can collect in pockets of lower polymer density. These pockets can enlarge over time until they become discreet vacuoles of water visible as glistenings or subsurface nanoglistenings [4]. Vacuoles with diameters from less than 200 nm located up to 120 μm below the surface of the IOL are called subsurface nanoglistenings [5].

The Acrysof hydrophobic acrylic IOL material (Alcon, Fort Worth, USA) has become increasingly popular since the 1990s and is now a widely used IOL material that is approved by all regulatory authorities around the world. Since its introduction, increasing light scattering due to glistening formation has been observed in lenses made from Acrysof IOL material [6]. Miyata et al. introduced a clinical grading system based on the number of particles seen in slit-lamp examination [7]. To better study glistenings in-vitro, accelerated aging methods have been developed to intentionally generate ex-vivo glistenings [1]. Then, in accordance with the clinical grading system, IOLs can be divided into different glistening categories depending on the number of microvacuoles per square millimetre that are produced after the aging procedure. Using such methods, the impact of glistenings on the optical performance has been studied and is now well understood. Glistenings have a rather small effect on the central image quality; their impact on light scattering, on the other hand, is greater [8, 9].

We evaluated, using an established in vitro laboratory method, the formation of glistenings of a new hydrophobic IOL, one which the manufacturer claims is more resistant to glistening formation: the Eyecryl Plus ASHFY600, and compared it to the well-established and accepted AcrySof IQ SN60WF.

## Methods

### Intraocular lenses

Ten monofocal Eyecryl Plus ASHFY600 IOLs (Biotech Vision Care, Ahmedabad, India) and ten monofocal AcrySof IQ SN60WF IOLs (Alcon, Fort Worth, USA) were tested for their resistance to glistening formation. All IOLs had the same refractive power of + 21.0 dioptres. The Eyecryl Plus ASHFY600 IOL and AcrySof IQ SN60WF are both single-piece IOLs, made from a hydrophobic acrylic material (Table 1).

**Table 1:** Characteristics of the studied IOL materials

IOL model	Manufacturer	Optic Copolymer	Cross-Linker	Equilibrium Water Content (in percent)	Blue-Light Filter	Manufacturing process
Eyecryl Plus ASHFY600	Biotech	Phenylethyl acrylate (PEA) and phenylethyl methacrylate (PEMA)	n.d.	< 5%	Yes	Lathe-cut
AcrySof IQ SN60WF	Alcon	Phenylethyl acrylate (PEA) and phenylethyl methacrylate (PEMA)	butanediol diacrylate (BDDA)	0.1–0.5	Yes	Cast-moulding

*IOL* intraocular lens, *n.d.* not disclosed

### Accelerated aging

Microvacuoles (glistenings) were induced in-vitro by temperature changes using an established accelerated aging protocol as previously described in our earlier studies [8, 10]. In short, the lenses were hydrated in Sodium Chloride solution (0.9%) in glass flasks and stored in an oven at 45 °C for 24 h. After removal from the oven, the temperature was reduced to 37 °C by immersing the flasks in a water-bath. The lenses were kept at 37 °C for 2.5 h.

### Evaluation of Glistenings

**Fig. 1:**
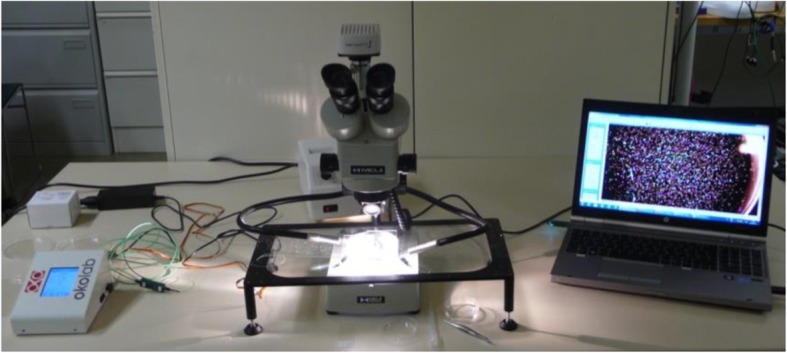
Setup for evaluation of glistenings. Left to right: Heated stage used to maintain and monitor the temperature during glistening evaluation; Microscope over a Petri dish including an IOL under test on an illuminated, heated plate; Laptop with image analysis software

**Fig. 2:**
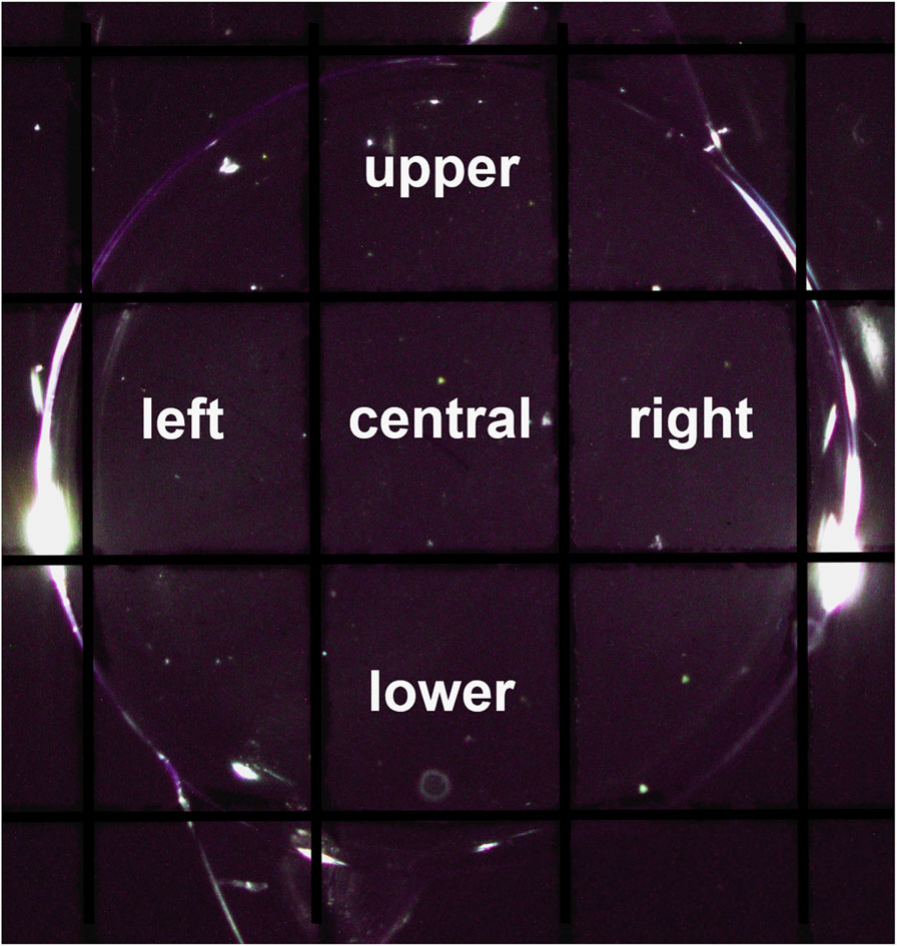
Intraocular lens optic sectioned by a standard grid. In all IOLs, 5 sections of the lens optic were analysed (central, left, upper, right, lower)

All samples were examined under an EMZ-8TR Trinocular Zoom Stereo microscope (Meiji Techno, Saitama, Japan). Microscopic images of all IOLs were taken immediately after aging process using an Infinity-2CB digital camera (Lumenera, Nepean, Canada) (Fig. 1). After placing a grid behind the IOL that separates the lens optic into five standardized rectangular sections, an overview image in 14-fold magnification was obtained of the whole optic as a qualitative overview image (Fig. 2). Using 90-fold magnification, an image was made for each section: the central section and four peripheral sections: to evaluate the number of glistenings in each section.

**Fig. 3:**
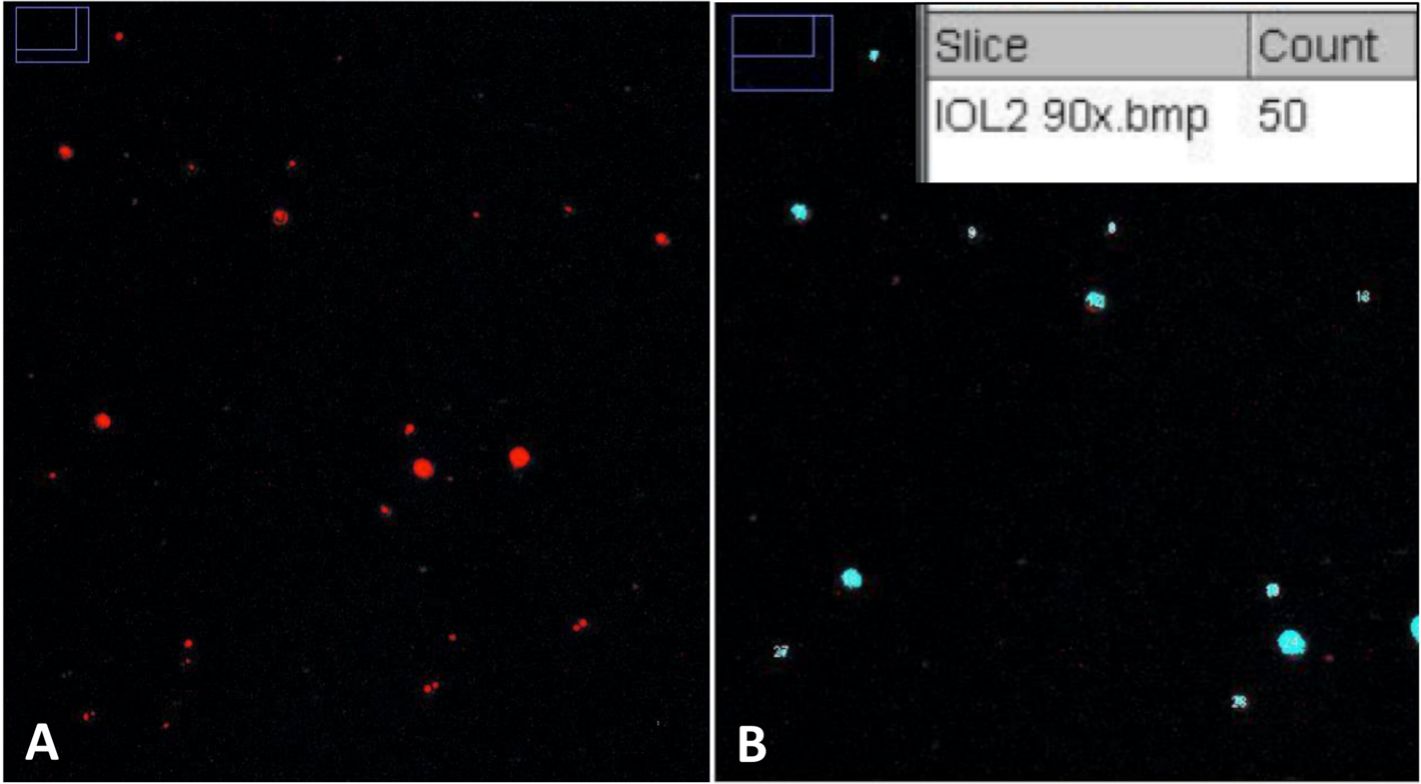
Binary transformed exemplary images. **a** Saturation and Brightness were adjusted and a color threhold technique was applied to separate glistening particles (red) from the background (black). **b** Counting of the glistenings (here blue) was performed automatically by an image analysis software (ImageJ, 1.49v) [11]

Image analysis was performed using the ImageJ software 1.49v. [11] Prior to image analysis parameters for the median filter and automated thresholding have been predefined using test images with low to high glistening numbers. Investigators were blinded for the IOLs under test. Irregular optical fluctuations have been removed by a smoothing procedure using a nonlinear median filter. Contrast and brightness were optimized using the same settings for each IOL (Fig. 3a). An automated threshold technique was used with the predefined threshold value to separate image information in a binary image - to distinguish glistenings from the background. The software automatically counted the number of glistenings (Fig. 3b). Number of glistenings was evaluated for all five sections of the IOL optic. (Note, this approach is only suitable when the number of glistenings is moderate so that there is no overlapping of glistenings.)

A 1200 × 1600 pixels area of the images in 90-fold magnification was selected to evaluate the number of glistenings. The central section was observed to correspond to the region with the highest glistening density. An image of a micrometer in 90-fold magnification was used to calibrate results with the dimensions of the lens to determine the density of glistenings. As 1 mm corresponds to 1086 Pixels and the original image size was 1200 Pixels × 1600 Pixels, total image size was 1.63 mm^2^. Given number of glistenings was divided by 1.63 to obtain the number of microvacuoles per square millimetre (MVs/mm^2^).

The number of glistenings of the central part of the lenses was compared to a modification of the Miyata scale [7]: Grade 0 (< 25 MVs/mm^2^), grade 1 (25–100 MVs/mm^2^), grade 2 (100–200 MVs/mm^2^), grade 3 (> 200 MVs/mm^2^).

### Data analysis

The number of MVs/mm^2^ in the central part and from all five sections was averaged for ten IOLs from each group and given as mean (±standard deviation). Statistical analysis was performed using Excel V.14.7.7 (Microsoft Corporation, Redmond, USA) performing two-sided student’s t-tests. A *P*-value less than 0.05 was considered statistically significant.

## Results

### Material purity

**Fig. 4:**
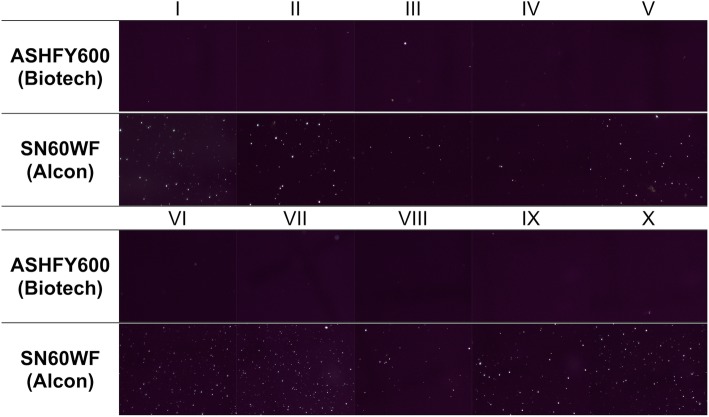
Microscopic images of the central part of all tested IOLs. Images were obtained under a microscope in a 90-fold magnification after standardized accelerated glistening induction

Images of the central part of the lens in 90-fold magnification show only a few glistenings in the Eyecryl Plus ASHFY600 with low variability between all ten Eyecryl IOLs. A larger number of glistenings was observed in the AcrySof IQ SN60WF IOLs (Fig. 4). Software image analysis revealed that the number of microvacuoles per square millimetre was highest in the central part of the AcrySof IQ SN60WF IOL with 41.84 (±27.67) MVs/mm^2^. The lowest amount of glistenings was obtained averaging the five sections of the Eyecryl Plus ASHFY600, with 0.52 (±0.24) MVs/mm^2^. For the AcrySof IOLs the glistening number in the central part was higher compared to the value of all 5 sections (*p* <  0.05), for the ASHFY600 both values were very similar, without a statistically significant difference (*p* = 0.32) (Table 2).

**Table 2:** Density of glistenings. Comparison of the mean values of the two studied intraocular lens models

	central part			mean of 5 sections		
IOL	Eyecryl	AcrySof	*p-value*	Eyecryl	AcrySof	*p-value*
Average MV/mm^2^ (± standard deviation)	0.7 (±0.5)	41.8 (±27.7)	< 0.05*	0.5 (±0.2)	19.9 (±10.6)	< 0.05*

*IOL* intraocular lens, *MV/mm*^*2*^ microvacuoles per square millimetre, *student’s t-test

### Miyata grading

**Fig. 5:**
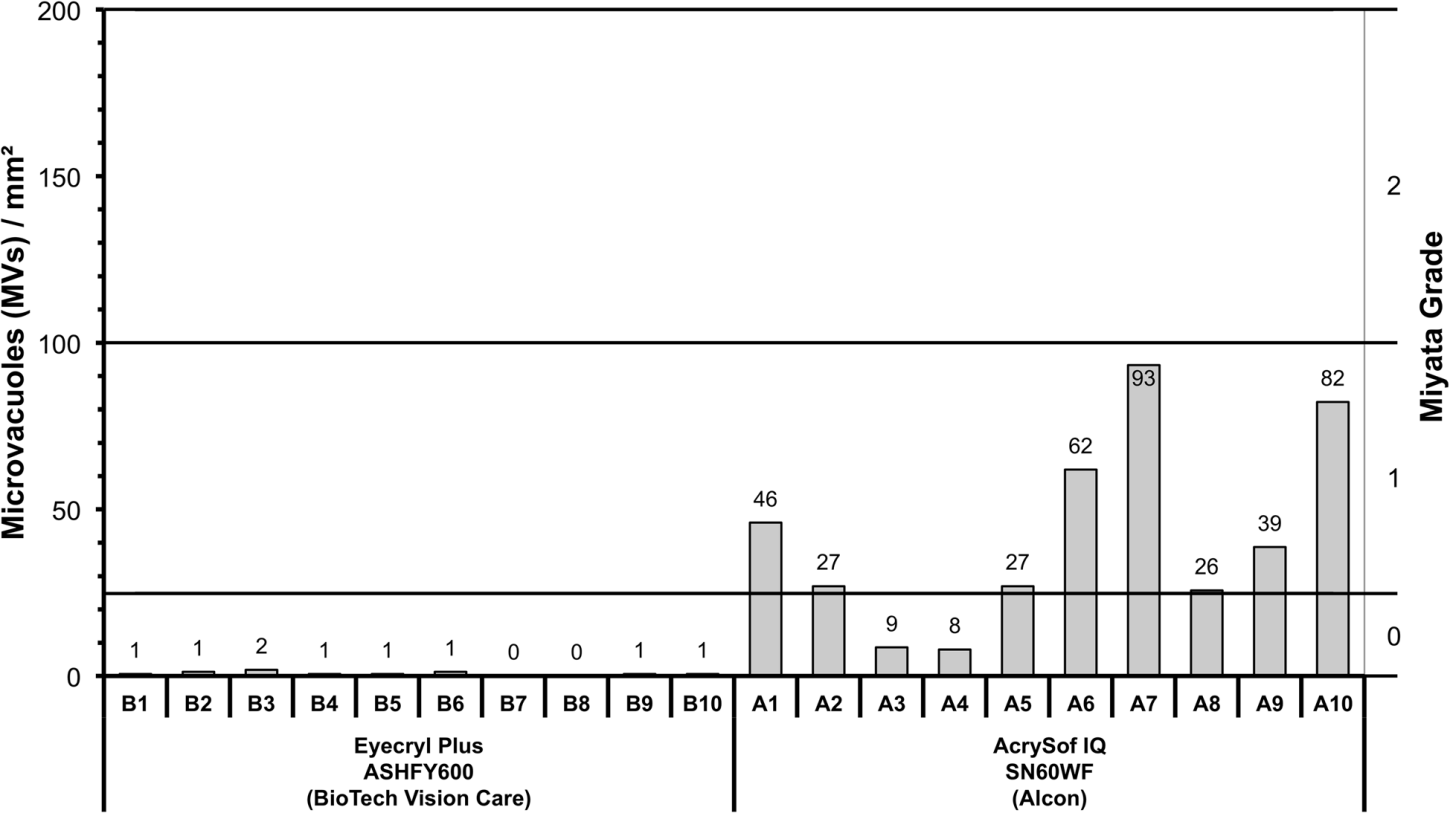
Number of glistenings in the central part of all tested IOLs after accelerated glistening induction. The secondary y-axis shows the relationship to the Miyata grading system. MVs/mm^2^, microvacuoles per square millimetre

All of the Eyecryl Plus ASHFY600 IOLs were classified as Miyata Grade 0. Three of ten AcrySof IQ SN60WF IOLs reached Miyata grade 1 but none of them scored Miyata grade 2 (Fig. 5).

## Discussion

The Eyecryl ASHFY600 IOL showed high resistance to glistening formation using an established laboratory accelerated aging model. Furthermore, compared to the well-established AcrySof SN60WF, the ASHFY600 had a lower mean glistening grade. In general, glistening numbers were higher in the central part of the lens compared to the periphery in the AcrySof IOLs, corresponding to the lens thickness, which is highest in centre of the IOL optic. Due to the overall low number of glistenings in the ASHFY600 IOLs, mean values for the central section and the periphery did not differ significantly (0.7 and 0.5, respectively).

In general, hydrophobic acrylate has some advantages over other IOL materials. Lenses made of hydrophobic acrylate show a lower tendency to develop posterior capsule opacification in comparison to those made of polymethyl methacrylate (PMMA) or hydrophilic acrylate [12]. Complications associated with hydrophilic acrylate lenses like IOL calcification have not been described in hydrophobic IOL material [13]. Hydrophobic acrylate IOLs can be cost-effectively produced and offer good handling during small incision cataract surgery [4].

Despite these benefits, hydrophobic acrylic IOLs are prone to develop glistenings. This long-term change in the material can worsen the lens’ optical performance [8, 9]. In recent research, our group has examined the nature of this deterioration in vision that is attributable to glistenings. Our colleagues, Weindler et al. demonstrated that a large number of glistenings is needed to affect the central image quality [8]. They induced varying amounts of glistening in monofocal AcrySof IOLs and evaluated glistenings’ impact on the image quality by measuring the lenses’ modulation transfer function (MTF) and Strehl ratios. The MTF value was reduced from 0.580 in clear control lenses to 0.533 in lenses with over 500 MV/mm^2^ at a special frequency of 100 lp/mm and a 3-mm-aperture [8]. Thus, glistenings have a rather small effect on the central image quality but their main effect is in changing another optical performance parameter, as a recent study by our group has shown. Labuz et al. found that straylight increases proportionally to the number of microvacuoles per square millimetre. Glistenings were induced in six different hydrophobic IOL models. IOLs with a mean central number of 3532 MV/mm^2^ showed elevated straylight levels of 19.3 deg^2^/sr, which would result in difficulties for patients while driving [9, 14]. Fortunately, in the presented study, mean glistening numbers were lower in both of the IOL models under test, suggesting improvements in these hydrophobic materials.

In 2013, Thomes and Callaghan reported on the continuous improvements (for which they unfortunately do not provide details) in manufacturing process of the Acrysof copolymer intended to reduce the incidence of glistening formation. They compared AcrySof lenses manufactured in 2003 with those made in 2012 [1]. The 2012 manufactured AcrySof demonstrated a significant reduction in glistening number (39.9 ± 35.0 MV/mm^2^) compared to lenses produced in 2003 (315.7 ± 149.4 MV/mm^2^). Our results showed similar values for Acrysof produced in 2017, with a mean number of central glistenings of 41.84 (±27.67) MVs/mm^2^ suggesting a maintenance of the improved process that leads to the reduced glistening formation.

The Eyecryl ASHFY600 IOL is made from a hydrophobic acrylate polymer (Table 1).

The Eyecryl lens is manufactured by lathe-cutting the polymer which is different to the way Acrysof IOL is made, which is cast-moulding manufactured. Possibly the Eyecryl lens retains a more homogenous copolymer distribution within the final IOL whereas the cast-moulding procedure of the Acrysof lens might be rearranging the polymer distribution. In cast-moulding, care must be taken to avoid the development of inhomogeneities that can re-distribute co-polymers, chances which would make the lens susceptible to further material changes such as microvacuole formation [15]. In a comparative clinical study, Nishihara et al. found that lathe-cut lenses show better long-term stability (regarding surface light-scattering) compared to cast-moulded lenses [15].

After shaping the lens by lathe-cutting or cast-moulding, a subsequent step in manufacturing usually includes a polishing process. This stage has been shown to be the potential cause of postoperative material changes in hydrophilic acrylic lenses from a series of lenses affected by opacification, the residual polishing materials, like Aluminium Oxide, might have remained on the lens surface and provoking the postoperative clouding of the lenses [16].

Thus, the IOL production process as well as the polymer are crucial elements in providing a lens with a resistance to material changes. Our results suggest that lathe-cutting a lens is superior to cast-moulding and we consider the new technologies, such as laser-cutting the lens, might further improve IOL manufacturing.

Another approach to reduce the tendency for glistening formation is to improve the polymer by introducing hydrophobic IOL polymer compositions that have increased hygroscopy. Hygroscopy describes a material’s ability to absorb and hold water inside the material. Water entering the material connects with the hydrophilic groups, thus avoiding water accumulation in vacuoles or pockets and forming glistenings [4]. The more hygroscopic a material is, the higher its equilibrium water content (EWC) under certain environmental conditions. Apart from the composition of the material, the EWC depends on the concentration of salts in its surrounding solution and the environmental temperature. Early hydrophobic materials for IOLs had low hygroscopy: the AcrySof material introduced in the 1990s has an EWC as low as 0.1–0.5% [17]. Some of the new generation hydrophobic materials incorporate a certain amount of acrylate with hydrophilic groups, thus leading to equilibrium water contents around 4 to 5% [4]. Only a few companies disclose the exact copolymer composition used for their IOLs. One known composition is that of the enVista IOL made by Bausch & Lomb (New York, USA). Its copolymer consists of 3 different monomers: poly(ethylene glycol) phenyl ether acrylate (40%), 2-hydroxyethyl methacrylate (HEMA, 30%) and styrene (26%), cross-linked by ethylene glycol dimethacrylate (4%) - collectively called PHS copolymer. Due to the hydrophilic groups of the HEMA the material has a higher EWC of about 4% and shows a low tendency towards formation of glistenings [4]. Another new generation hydrophobic polymer formulation by PhysIOL (Liège - Belgium) also contains an (undisclosed) amount of a hydrophilic monomer to provide an equilibrium water content of 4.9%, again this offers a low tendency for glistening formation [18].

As described above, even though improvements in the Acrysof material between 2003 and 2012 led to an increasing resistance to glistening formation, one can still induce glistenings in these lenses [1]. Glistenings - even a low number of them is considered undesirable, and the Alcon company recently introduced a new material, named Clareon, that is considered to show minimal tendency towards glistening formation. The company does not disclose its exact material composition; Clareon’s EWC is around 1.5%. Several other IOL manufactures that have now addressed the problem of glistenings in their hydrophobic acrylic intraocular IOL materials: Vivinex (Hoya, Singapore), Tecnis (Johnson&Johnson, New Jersey, USA) and RayOne (Rayner, Hove, UK). In our laboratory, in-vitro accelerated aging studies have confirmed that lenses made of these materials and the Eyecryl ASHFY600 IOL are “glistening-free”. As this study was conducted in an in-vitro environment, results cannot be transitioned to the clinic without restriction. Therefore, long-term clinical studies have to confirm the lower amount of glistenings in IOLs made of advanced hydrophobic materials.

## Conclusion

The new Eyecryl ASHFY600 IOL has low tendency towards glistening formation. With a mean value of 0.52 (±0.24) MV/mm^2^ all over the IOL and 0.74 (±0.54) MV/mm^2^ in its central part after accelerated aging, the corresponding grade on the Miyata Scale was 0 for all tested lenses. Resistance against glistening formation was superior to the well-established AcrySof IQ SN60WF IOL, which in comparison showed values of 19.89 (±10.57) MV/mm^2^ all over the IOL and 41.84 (±27.67) MV/mm^2^ in the centre of the lens optic.

## Data Availability

The datasets used and/or analysed during the current study are available from the corresponding author on reasonable request.

## Abbreviations

IOL: Intraocular lens
MV: microvacuoles
PMMA: polymethyl methacrylate
MTF: modulation transfer function
EWC: equilibrium water content
HEMA: hydroxyethyl methacrylate
PHS: phenyl ether acrylate, hydroxyethyl methacrylate, styrene
